# Dietary Enrichment with Fish Oil Prevents High Fat-Induced Metabolic Dysfunction in Skeletal Muscle in Mice

**DOI:** 10.1371/journal.pone.0117494

**Published:** 2015-02-06

**Authors:** Lisa K. Philp, Leonie K. Heilbronn, Alena Janovska, Gary A. Wittert

**Affiliations:** Discipline of Medicine, University of Adelaide, Adelaide, South Australia, Australia; Faculty of Biology, SPAIN

## Abstract

High saturated fat (HF-S) diets increase intramyocellular lipid, an effect ameliorated by omega-3 fatty acids *in vitro* and *in vivo*, though little is known about sex- and muscle fiber type-specific effects. We compared effects of standard chow, HF-S, and 7.5% HF-S replaced with fish oil (HF-FO) diets on the metabolic profile and lipid metabolism gene and protein content in red (soleus) and white (extensor digitorum longus) muscles of male and female C57BL/6 mice (n = 9-12/group). Weight gain was similar in HF-S- and HF-FO-fed groups. HF-S feeding increased mesenteric fat mass and lipid marker, Oil Red O, in red and mixed muscle; HF-FO increased interscapular brown fat mass. Compared to chow, HF-S and HF-FO increased expression of genes regulating triacylglycerol synthesis and fatty acid transport, HF-S suppressed genes and proteins regulating fatty acid oxidation, whereas HF-FO increased oxidative genes, proteins and enzymes and lipolytic gene content, whilst suppressing lipogenic genes. In comparison to HF-S, HF-FO further increased fat transporters, markers of fatty acid oxidation and mitochondrial content, and reduced lipogenic genes. No diet-by-sex interactions were observed. Neither diet influenced fiber type composition. However, some interactions between muscle type and diet were observed. HF-S induced changes in triacylglycerol synthesis and lipogenic genes in red, but not white, muscle, and mitochondrial biogenesis and oxidative genes were suppressed by HF-S and increased by HF-FO in red muscle only. In conclusion, HF-S feeding promotes lipid storage in red muscle, an effect abrogated by the fish oil, which increases mediators of lipolysis, oxidation and thermogenesis while inhibiting lipogenic genes. Greater storage and synthesis, and lower oxidative genes in red, but not white, muscle likely contribute to lipid accretion encountered in red muscle. Despite several gender-dimorphic genes, both sexes exhibited a similar HF-S-induced metabolic and gene expression profile; likewise fish oil was similarly protective in both sexes.

## Introduction

Diets rich in saturated fat (HF-S), particularly when consumed *ad libitum*, increase adiposity in rodents [1, 2]. Additionally, HF-S diets increase triacylglycerol deposition in ectopic stores, including the liver and skeletal muscle in both mice [3–5] and humans [6–9]. We have previously reported [10] that *in vitro* exposure to palmitate, a saturated fatty acid, increases triacylglycerol content in rat L6 myotubes. In healthy lean humans, acute exposure to a HF-S diet increases fatty acid oxidation [11, 12]. This adaptive increase is impaired in obese humans [11, 13, 14] and has been linked to increased intramyocellular triacylglycerol storage [4, 5, 15, 16], although increased fatty acid uptake [17, 18] and reduced triacylglycerol hydrolysis [19, 20] may also be involved.

Dietary supplementation with fish oil has multiple, well-established health benefits, including reducing circulating triacylglycerol and an anti-inflammatory action [21, 22]. In mice, replacing 1.5% of a corn oil based high fat diet (35% lipid; n-6 PUFA-rich) with docosahexaenoic acid (DHA) derivative, α-ethyl DHA ethyl ester, reduced intramyocellular triacylglycerol by more than half [23] and likewise, incorporating 3.6% eicosapentaenoic acid (EPA) ethyl ester into a 45% high fat diet elicited intramyocellular triacylglycerol lowering effects. Mechanistically several pathways may be involved. A high fat diet (35.5% energy from fat) of predominantly fish oil increased muscle fatty acid transporter, *Fat/cd36*, mRNA in mice, compared to those consuming an isocaloric lard-based high fat diet [24]. Supplementing a cafeteria high fat diet (62% energy from fat (45% saturated fat, no EPA and DHA)) with EPA ethyl ester (1g/kg), reduced acetyl CoA carboxylase β (*Acc-β*) mRNA content in rat muscle, which may simultaneously result in suppression of lipogenesis and enhanced β-oxidation in myocytes [25]. While replacing 15% of fat in high fat diet (35.2% fat (mainly n-6 PUFA-rich corn oil)) with n-3 PUFA concentrate (3.29:1 DHA:EPA) in mice promoted efficient β-oxidation of fatty acids within skeletal muscle mitochondria [26]. Whether n-3 PUFAs from natural fish oil abrogate intramyocellular lipid accumulation in a HF-S setting is unclear and the pathways involved require clarification. Additionally, the vast majority of past studies have examined only the effect of dietary n-3 PUFA supplementation in either white glycolytic skeletal muscles [24] or muscles of mixed fiber type [25–30] that are reliant on glucose or a fuel mixture as their main substrate [31]. Red fibers are oxidative and rely heavily on lipids as fuel [31–33]. The extent to which the addition of n-3 PUFAs to a HF-S diet modifies lipid metabolism in these red fibers remains to be determined, although increased *Cpt1b* and *Ucp3* mRNA have been reported in the mouse soleus in response to EPA ethyl ester enrichment of a high fat diet [34]. Past studies were also conducted solely in male rodents and thus cannot answer whether there are sex-related differences in this response. Especially as in rats [35, 36], high fat-fed males are reported to be less efficient at promoting adipose tissue deposition, are less proficient at amplifying muscle oxidative capacity and instead exhibit greater hepatic triacylglycerol content and fatty acid oxidation rate, when compared to high fat-fed females [36]. The aim of this study was therefore to determine the differential effects of feeding a saturated fat-rich diet (HF-S) or a HF-S diet with 7.5% of fatty acids replaced with n-3 PUFAs from fish oil (HF-FO) in male and female mice on body composition and pathways of skeletal muscle lipid metabolism in both “white” fast-twitch glycolytic and oxidative-glycolytic and “red” slow-twitch oxidative muscles, by quantifying the histological changes in muscle lipid content and oxidative capacity (SDH, NADH-TR) and the mRNA content of key genes involved in fatty acid transport (*Fat/cd36*, *Fabp*_*pm*_, *Fatp1*, *Fatp4*), lipogenesis and triacylglycerol storage and hydrolysis (*Srebf1*, *Insig1*, *Dgat1*, *Scd1*, *Hsl*), and fatty acid disposal (*Pdk4*, *Ampk*α *1*, *Ampk*α *2*, *Acc-β*, *Cpt1b*, *Ucp3*, *Pgc1*α, *Ppar*α) using real time quantitative PCR and the abundance of key proteins involved in mitochondrial oxidation by Western blot analysis (PGC1α, PPARα, CPT1b, OXPHOS Complex I-V).

## Materials and Methods

### Ethics Statement

All procedures were approved by the University of Adelaide and Institute of Medical and Veterinary Science Animal Ethics Committees, and University of Adelaide guidelines for the use and care of laboratory animals were followed (approval number: M-027–2007). All animals were provided with environmental enrichment throughout the dietary intervention and all procedures thereafter were performed under isoflurane-induced anaesthesia to minimise suffering.

### Animals and diets

Specific pathogen-free 6-week-old male and female C57BL/6J mice were purchased from Laboratory Animal Services, University of Adelaide (Adelaide, Australia). Mice were housed in individual cages in an animal holding room with fixed photoperiod (12:12hr light/dark cycle) and temperature (24.5°C). On arrival, mice underwent an acclimatization period of 2 wks, during which they were provided standard rodent chow diet and water *ad libitum*. Following the acclimatization period, mice were randomly assigned to one of three diets, fed either a standard chow ((control (CON); AIN-93G), 16.1 MJ/kg, 15.91% energy from fat, 25.08% energy from protein, 58.48% energy from carbohydrate), high saturated fat ((HF-S; SF07–066), 21.8 MJ/kg, 59.60% energy from fat (rich in saturated fat), 18.53% energy from protein, 21.20% energy from carbohydrate), or HF-S fish oil enriched ((HF-FO; SF07–067), 21.8 MJ/kg, 59.60% energy from fat (7.5% of HF-S replaced with n-3 PUFAs (% as fed)), 18.53% energy from protein, 21.27% energy from carbohydrate) diet. Diets were manufactured by Specialty Feeds Pty Ltd (Glen Forrest, Australia) and n-3 PUFAs in the HF-FO diet were provided as HiDHA 25N tuna oil (26% DHA, 6% EPA) (kindly donated by Nu-mega Ingredients Pty Ltd (Nathan, Australia)). Both high fat diets were stored at -20°C, whilst the HF-FO diet was also stored in aliquots under nitrogen gas to avoid oxidation. Mice were maintained on their respective diets for a period of 11 or 14 wks, see Experimental Protocols and Tissue Collection, during which food and water were provided daily *ad libitum*.

### Experimental Protocols and Tissue Collection

Cohort 1: Mice were maintained on their respective diets for 14 wks (± 4 d). Body weight (g) was measured thrice weekly and at the time points of arrival (6 wks-of-age) and post-mortem (20 wks-of-age) (n = CON(male) = 10, CON(female) = 12, HF-S(male) = 11, HF-S(female) = 11, HF-FO(male) = 9, HF-FO(female) = 11). Body weight upon arrival (Table 1; *P* = 0.16 (CON vs. HF-S), *P* = 1.0 (CON vs. HF-FO), *P* = 0.99 (HF-S vs. HF-FO) and during the acclimatization period (data not shown; *P* = 0.24 (CON vs. HF-S), *P* = 0.42 (CON vs. HF-FO), *P* = 1.0 (HF-S vs. HF-FO)) was similar in all dietary groups. Food intake was measured daily, from which cumulative energy intake (MJ) was calculated.

**Table 1:** Body weight-related and plasma biochemical parameters.

	Male			Female			Stat
	CON	HF-S	HF-FO	CON	HF-S	HF-FO	
Start Weight (g) ^†^	22.0 ±0.6	22.5 ±0.2	22.6 ±0.3	17.9 ±0.2	18.9 ±0.3	18.3 ±0.3	S
Final Weight (g) ^†^	28.0 ±0.8	31.1 ±0.7*	31.8 ±0.9*	21.9 ±0.3	26.0 ±0.8*	27.6 ±0.6*	D, S
Weight Gain (g) ^†^	6.05 ±0.8	8.63 ±0.6*	9.25 ±0.9*	3.99 ±0.3	7.14 ±0.7*	9.25 ±0.6*	D, S
Energy Intake (MJ)	4.36 ±0.1	5.45 ±0.1^§^	5.08 ±0.2^§^	3.94 ±0.1	5.50 ±0.2^§^	5.49 ±0.1^§^	DxS
Pooled AT (g)	2.48 ±0.2	3.00 ±0.3*	3.19 ±0.5*	1.64 ±0.1	2.64 ±0.4*	2.92 ±0.3*	D
Subcutaneous AT (mg/g)	35.0 ±2.4	40.5 ±4.8	41.5 ±6.3	34.0 ±2.1	44.9 ±5.4	47.3 ±6.0	N/S
Mesenteric AT (mg/g)	20.0 ±1.0	18.7 ±0.6	15.6 ±1.1 ^#^	17.9 ±0.6	21.7 ±2.2	18.4 ±0.8 ^#^	D
Perirenal AT^†^ (mg/g)	8.2 ±0.7	9.3 ±1.1	11.5 ±2.0*	5.9 ±0.4	7.8 ±1.0	9.9 ±1.0 *	D, S
Brown AT (mg/g)	4.7 ±0.4	3.8 ±0.3	5.2 ±0.5 ^#^	4.4 ±0.4	3.8 ±0.2	5.5 ±0.4 ^#^	D
Perigonadal AT (mg/g)	20.0 ±1.3	22.3 ±2.4	23.8 ±4.0	13.4 ±1.4	18.6 ±2.3	22.4 ±2.9*	D
Plasma Glucose (mM) ^†^	8.04 ±0.6	7.98 ± 0.5	6.84 ±0.7	7.34 ±0.6	5.96 ±0.5	6.64 ±0.5	S
Plasma Insulin (pM)	63.8 ±12	109.7 ±29	59.7 ±14	71.9 ±14	114.2 ±26	70.7 ±18	N/S
Plasma TG (mM)	0.83 ±0.1	0.62 ±0.0	0.53±0.1* ^#^	0.72 ±0.1	0.79 ±0.1	0.55±0.1* ^#^	D

Measured in male and female mice fed control (CON), high saturated fat (HF-S) and high fat fish oil enriched (HF-FO) diets for 14 wks (Cohort 1).

Results are mean ± SEM of 9–12 animals per group. Adipose tissue, AT; TG, triacylglycerol.

Statistics: Effect of diet (D):

*P≤0.05, vs CON;

^#^ *P*≤0.05, compared to HF-S. Effect of sex (S):

^†^ *P*≤0.05 male vs female. Diet*sex interaction (DxS):

^§^ *P*≤0.05, compared to CON of same gender. N/S, not significant.

Following 14 wks diet, whilst in the fed-state, mice underwent non-recovery surgery for the excision of skeletal muscle. Surgeries were scheduled and performed to minimise temporal variation. Anaesthesia was induced using a mixture of oxygen (0.5 L/min), nitrous oxide (0.5 L/min) and 2% isoflurane (Forthane, Abbott Australasia Pty Ltd (Kurnell, Australia)) and maintained using 1.5% isoflurane, 0.4 L/min oxygen and 0.4 L/min nitrous oxide. The whole “white” extensor digitorum longus (EDL) and “red” soleus (SOL) muscles were then rapidly dissected, snap frozen in liquid nitrogen and stored under liquid nitrogen vapour phase storage until subsequent analyses of mRNA content and protein abundance. Following skeletal muscle surgery, a cardiac puncture was performed and blood was collected in Microvette CB300 tubes treated with EDTA dipotassium salt (Sarstedt (Nümbrecht, Europe)). Post-mortem, adipose tissue from the pooled posterior and anterior subcutaneous (dorso-lumbar, inguinal, gluteal, white interscapular, subscapular, axillo-toracic and superficial cervical depots); visceral mesenteric; pooled perirenal and retroperitoneal; pooled brown deep cervical and interscapular; and perigonadal (periovariac, females; epididymal, males) depots was dissected, snap frozen in liquid nitrogen and evaluated for weight. Adipose tissue depot weights are expressed relative to body weight, to allow comparison across sexes.

Cohort 2: Mice were maintained on their respective diets for 11 wks (± 7 d). Following 11 wks diet, whilst in the fed-state, mice underwent non-recovery surgery for the excision of skeletal muscle, with anaesthesia induced and maintained as described above. The whole EDL (undivided from the tibialis muscle), and soleus (undivided from the gastrocnemius and plantaris muscles) were then rapidly dissected, followed by excision of the whole undivided quadriceps muscles. Muscle groups were embedded at resting tension in Tissue-Tek OCT (Sakura Finetek Co Ltd (Tokyo, Japan)) and gently frozen. OCT-embedded muscles were stored under liquid nitrogen vapor phase storage until subsequent histological analyses of muscle lipid, fiber type composition and oxidative capacity.

### Plasma Biochemistry

Plasma glucose and triacylglycerol (mM) concentrations were measured in duplicate using Gluco-quant Glucose/HK and TG GPO-PAP kits (Roche Diagnostics (Mannheim, Germany)), respectively, on the COBAS Bio automated analysis system (Roche Diagnostics Australia Pty Ltd (Castle Hill, Australia)). Plasma insulin concentrations (pM) were measured by DRG Ultrasensitive Mouse Insulin ELISA (DRG Instruments (Marburg, Germany)) as per manufacturer’s instructions.

### mRNA Expression Analyses using the GenomeLab GeXP Genetic Analysis System

Total RNA from whole EDL and soleus muscles was isolated using TRIzol reagent (Invitrogen Australia Pty Ltd (Mount Waverley, Australia)). RNA quality and concentration were evaluated (NanoDrop 1000 Spectrophotometer, ThermoScientific Inc (Wilmington, USA)). RNA was treated using DNase I (Invitrogen Australia Pty Ltd). cDNA was generated by reverse transcription according to manufacturer’s instructions (GenomeLab GeXP Genetic Analysis System, Beckman Coulter Inc), resulting in cDNA of gene-specific sequences with a flanking universal sequence. Forward and reverse primers were designed to amplify a section of the protein coding sequence and to be positioned in different exons as determined by Entrez Nucleotide (National Center for Biotechnology Information (Bethesda, USA)) and Ensembl (European Bioinformatics Institute/Wellcome Trust Sanger Institute (Cambridge, United Kingdom)), respectively. Primers were designed using GenomeLab GeXP eXpress Profiler software (ver.10.0 Beckman Coulter Inc (Fullerton, USA)) to generate an amplified product with a gene fragment length between 137–356 nucleotides and separation size of at least 6 nucleotides. Finally primer sequences were submitted to BLAST (Basic Local Alignment Search Tool, National Center for Biotechnology Information (Bethesda, USA)). A universal sequence was then added to both forward and reverse designed primer sequences, generating chimeric primers. Resultant forward and reverse primers (GeneWorks Pty Ltd (Hindmarsh, Australia)) are listed in table form (Supporting Information—S1 Table). Primers were optimized in singlet and multiplex reactions, according to manufacturer’s instructions (GenomeLab GeXP Genetic Analysis System, Beckman Coulter Inc). Multiplex PCR was then performed on the Eppendorf Mastercycler Gradient (Eppendorf South Pacific Pty Ltd (North Ryde, Australia)). Negative controls (no template, no reverse transcriptase) were run in parallel. Fluorescently-labeled PCR products were separated, detected, quantified and analyzed in duplicate using the GenomeLab GeXP Genetic Analysis system and GenomeLab GeXP eXpress Profiler software. Output mRNA contents were then normalized to the average mRNA content of 3 housekeeping genes, TATA-binding protein, RNA Polymerase 2c and large ribosomal protein P0.

### Protein content analyses by Western Blot

Whole EDL and soleus muscles were weighed and homogenised in ice-cold lysis buffer as described previously [37] and protein concentration determined by bicinchoninic acid protein assay (Pierce Biotechnology (Rockford, USA)). For measuring the abundance of PPARα, oxidative phosphorylation (OXPHOS) complexes I-V, peroxisome proliferative activated receptor γ coactivator 1α (PGC1α), CPT1b and β-tubulin proteins, 16–20 μg of muscle protein was subjected to SDS-PAGE using precast 10% Bis-Tris gels or 4–12% Bis-Tris gels (Bio-Rad Laboratories Pty Ltd (Gladesville, Australia)) and transferred to PVDF membranes. Membranes were incubated overnight in primary antibody; PPARα (Abcam (Cambridge, UK)), 1:500; OXPHOS proteins (MitoSciences (Eugene, USA)), 1:250; PGC1α (Abcam), 1:1000; CPT1b (Alpha Diagnostic International Inc (San Antonio, USA)), 1:1000; and β-Tubulin (Cell Signaling Technology (Danvers, USA)), 1:2000. Bound primary antibodies were detected with sheep anti-rabbit (1:2500, Chemicon International (Billerica, USA)) or goat anti-mouse alkaline-phosphatase linked antibody (1:2000, Millipore (Billerica, USA)). Membranes were developed with chemifluoresence substrate (ECF), scanned by Typhoon Imager (GE Healthcare Bio-Sciences (Rydalmere, Australia)) and were quantified using ImageQuant software (Molecular Dynamics).

### Muscle Histology

OCT-embedded muscle groups (the EDL and tibialis group, and the soleus, gastrocnemius and plantaris group, and the quadriceps muscles group) were cut to 4, 9 or 10 μm thick cross-sections at -20°C. Sections were stained with haematoxylin and eosin to confirm normal cellular morphology (data not shown) or with Oil Red O, a marker of intramyocellular lipid content. Serial sections were stained for myofibrillar myosin adenosine triphosphatase (mATPase), following alkaline (pH 10.4) and acidic (pH 4.1, pH 4.3) preincubations, to evaluate muscle fiber type [38]. Sections were stained for succinic dehydrogenase (SDH) and NADH tetrazolium reductase (NADH-TR) to confirm muscle fiber type and evaluate oxidative capacity. Following all staining, slides were scanned using the NanoZoomer Digital Pathology scanner (Hamamatsu Photonics K. K. (Hamamatsu City, Japan)). The soleus and EDL muscles were analyzed in duplicate to determine muscle fiber type and oxidative capacity in predominantly red and white muscles, respectively, with the same 75 muscle fibers in each scanned image assessed for all stains. Images were scored whilst blinded to dietary group and sex.

The activity of mATPase was judged on a subjective basis by one observer and muscle fibers were given a score from 1–5 based on the intensity of staining (1 = light, 2 = light-moderate, 3 = moderate, 4 = moderate-dark, 5 = dark). A defined scheme was used to classify fiber type, based on scores given to muscle fibers stained for mATPase [38]. Fiber type composition (%) was calculated as the proportion of each fiber type relative to the total number of fibers scored.

SDH and NADH-TR staining intensity were assessed by mean pixel density using ImageJ software (ver. 1.42q, National Institute of Health (Bethesda, USA)) from grayscale images manually traced at cell perimeters. The same fibers used to assess the activity of both SDH and NADH-TR were also used to determine muscle fiber type by mATPase staining, allowing classification of oxidative activity respective to fiber type. Cell area of each muscle fiber type was also assessed. The area of each fiber type was calculated relative to the total area measured, providing the relative area occupied by each muscle fiber type within the EDL and soleus muscles. Results are presented as the mean cross-sectional cell area of classified fibers and the % area occupied by each muscle fiber type.

### Statistical Analysis

All data are presented as mean ± SEM. Two-way ANOVA, with pairwise comparisons (Bonferroni post-hoc), was used to determine the effect of diet (CON, HF-S, HF-FO), muscle (EDL, soleus), sex (male, female) and their interaction on parameters. In Cohort 2 analyses, as there was no significant interaction of sex and diet, to enhance statistical power, data from male (n = 3 per group) and female (n = 2–3 per group) mice were combined. One-way ANOVAs, with Bonferroni post-hoc analysis, were therefore used to determine the effect of diet in the EDL and soleus muscles on muscle fiber type-specific parameters. Statistics were performed using Statistical Package for Social Scientists ver.17.0.0 (SPSS Inc (Chicago, USA)). *P*<0.05 was considered statistically significant.

## Results

### Effect of dietary fat composition on adipose tissue distribution and plasma biochemistry (Table 1)

Compared to CON, HF-S- and HF-FO-fed mice gained more weight (effect of diet: *P*≤0.001), but there were no differences between the two high fat diet groups. Irrespective of diet, males gained more weight than females (effect of sex: *P*≤0.05). Compared to CON, cumulative energy intake was higher in the two high fat diet groups (effect of diet: *P* <0.005), but there was no difference between high fat diets and no effect of sex. Compared to CON, HF-S and HF-FO increased pooled adipose tissue mass similarly, in both sexes (effect of diet: *P*≤0.05, *P*≤0.01). However, fat storage in distinct adipose tissue depots was affected by dietary fatty acid composition. Compared to CON, HF-FO increased pooled perirenal and retroperitoneal adipose tissue (effect of diet: *P*≤0.005), irrespective of sex, and gonadal periovariac adipose tissue (effect of diet: *P*≤0.05) in female mice. In males, gonadal depot, epididymal adipose tissue, was unchanged by diet; similarly subcutaneous fat mass was not diet-dependent. Compared to HF-S, HF-FO decreased mesenteric adipose tissue (effect of diet: *P*≤0.05) and increased brown adipose tissue (effect of diet: *P*≤0.05), in both sexes. Other than higher pooled perirenal and retroperitoneal adipose tissue in males (effect of sex: *P*≤0.05), the adipose tissue depots measured were not sexually dimorphic. Compared to CON and HF-S, HF-FO lowered plasma triacylglycerol (effect of diet: *P*≤0.005, *P*≤0.05), irrespective of sex. Neither high fat diet influenced plasma glucose nor insulin concentrations, irrespective of sex. Irrespective of diet, glucose levels were higher in males than females (effect of sex: *P*≤0.05).

### Effect of dietary fat composition on skeletal muscle lipid content in whole muscle and muscle fiber type distribution (Fig. 1)

**Fig 1:**
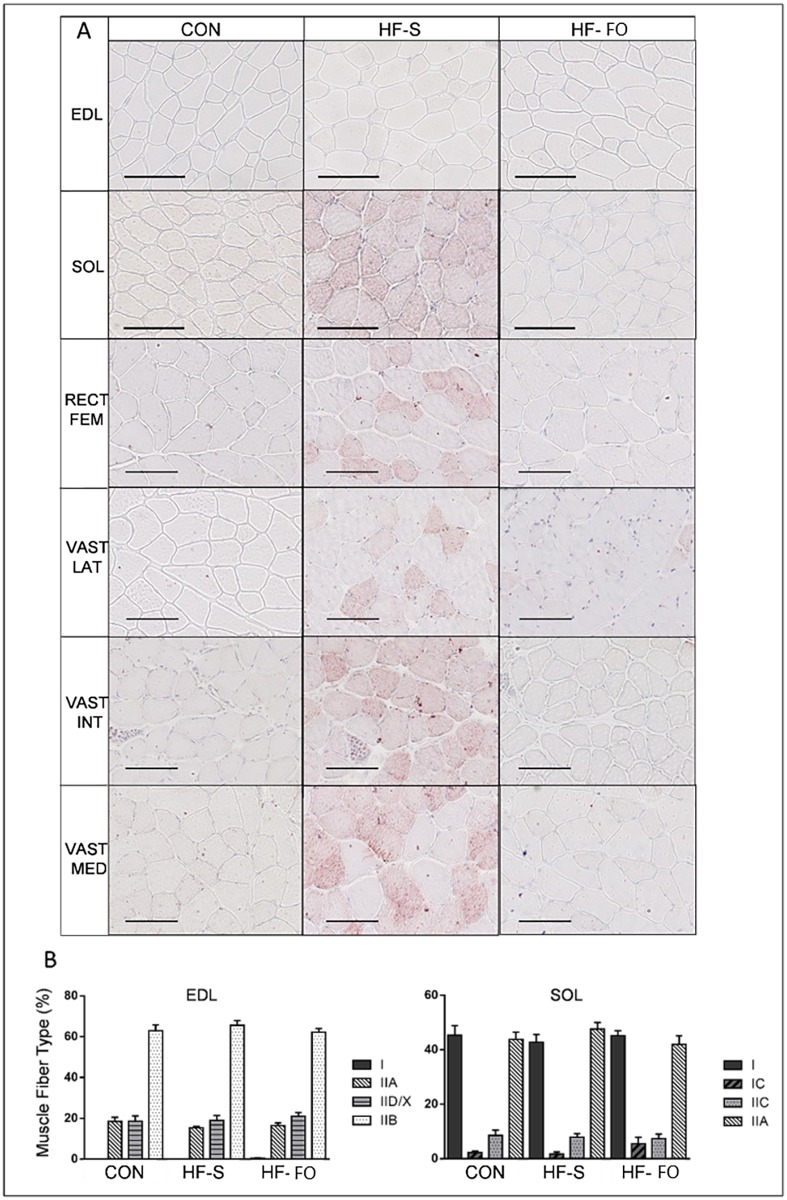
Muscle Lipid Content and Fiber Type Composition. (A) Oil Red O staining, a marker of intramyocellular lipid, of the whole extensor digitorum longus (EDL), soleus (SOL) and quadriceps (rectus femoris (RECT FEM), vastus lateralis (VAST LAT), vastus intermedius (VAST INT) and vastus medialis (VAST MED)) muscles and (B) muscle fiber type composition (%) of the EDL and SOL muscles of mice fed a control (CON), high saturated fat (HF-S) or high fat fish oil enriched (HF-FO) diet for 11 wks (Cohort 2). Scale bars represent 100 μm.

Increased Oil Red O staining, a marker of intramyocellular lipid, was apparent in soleus (predominately red) and quadriceps (mixed fiber type) of HF-S, but not CON and HF-FO, mice (Fig. 1A). Neither high fat diet influenced Oil Red O staining in the EDL (predominantly white muscle). Percentage muscle fiber type composition in the EDL and soleus muscles was not influenced by diet (Fig. 1B), nor was the cross-sectional area of each muscle fiber type (data not shown).

### Effect of dietary fat composition on muscle fatty acid transporter mRNA content (Table 2)

Compared to CON, both high fat diets increased *Fat/cd36* and *Fatp1* mRNA (effect of diet: *Fat/Cd36*: *P*≤0.001; *Fatp1*: HF-S *P*≤0.005; HF-FO *P*≤0.001), but HF-FO alone increased *Fatp4* mRNA (effect of diet: *P*≤0.02), irrespective of muscle type. Compared to HF-S, HF-FO increased *Fat/Cd36* mRNA (effect of diet: *P*≤0.001), irrespective of muscle type. All fatty acid transporters exhibited greater mRNA content in the soleus muscle (effect of muscle fibre type: *P*≤0.001). Except for higher *Fatp1* mRNA in males compared to females in the EDL only (muscle*sex interaction: *P*≤0.001), there were no other sex differences in fatty acid transporter mRNA content.

**Table 2:** Fatty acid transporter mRNA content.

		Male			Female			Stat
		CON	HF-S	HF-FO	CON	HF-S	HF-FO	
Fat/Cd36	EDL	100 ± 6	116 ± 8*	133 ± 5* ^#^	95 ± 7	105 ± 5*	137 ± 7* ^#^	D, M
	SOL^+^	100 ± 3	121 ± 4*	146 ± 7* ^#^	94 ± 5	115 ± 6*	139 ± 7* ^#^	
Fabp_pm_	EDL	100 ± 3	107 ± 4	100 ± 3	106 ± 8	98 ± 4	107 ± 5	M
	SOL^+^	100 ± 3	96 ± 2	110 ± 5	101 ± 6	100 ± 7	113 ± 8	
Fatp1	EDL^‡^	100 ± 8	137 ± 8*	145 ± 9*	81 ± 10	102 ± 7*	113 ± 13*	D, MxS
	SOL^+^	100 ± 6	114 ± 8*	129 ± 6*	93 ± 9	112 ± 12*	141 ± 9*	
Fatp4	EDL	100 ± 6	99 ± 9	119 ± 13*	107 ± 12	93 ± 7	108 ± 8*	D, M
	SOL^+^	100 ± 5	128 ± 10	150 ± 6*	117 ± 12	142 ± 20	142 ± 14*	

Fatty acid translocase (Fat/Cd36), Fatty acid binding protein (Fabp_pm_) and Fatty acid transport proteins 1 (Fatp1) and 4 (Fatp4) in extensor digitorum longus (EDL) and soleus (SOL) muscles of male and female mice fed control (CON), high saturated fat (HF-S) or high fat fish oil enriched (HF-FO) diets for 14 wks (Cohort 1).

mRNA contents are expressed as a percentage of the value of male animals under CON diet. Results are mean ± SEM of 9–12 animals per group. Statistics: Two-way ANOVA: Effect of diet (D):

*P≤0.05, vs CON;

^#^ *P*≤0.05, compared to HF-S. Effect of muscle type (M):

^+^ *P*≤0.05 EDL vs SOL. Muscle*sex interaction (MxS):

^‡^ *P*≤0.05, male vs female (EDL).

### Effect of dietary fat composition on the mRNA content of genes influencing fatty acid storage and lipogenesis (Table 3)

Compared to CON, HF-S increased *Srebf1* and *Insig1* mRNAs (effect of diet: *P*≤0.02), irrespective of muscle fiber type, and increased *Dgat1* and decreased *Scd1* mRNA in the soleus muscle alone (diet*muscle interaction: *P*≤0.05; *P*≤0.005). Compared to CON, HF-FO increased *Dgat1* mRNA in both muscles (diet*muscle interaction: *P*≤0.001). Compared to both CON and HF-S, HF-FO decreased *Scd1* mRNA in both muscles (diet*muscle interaction: *P*≤0.02) and increased *Hsl* mRNA (effect of diet: *P*≤0.001, *P*≤0.05), irrespective of fiber type. Compared to HF-S alone, HF-FO lowered the mRNA content of *Insig1*, irrespective of muscle type (effect of diet: *P*≤0.001) and increased *Dgat1* mRNA in the soleus muscle (diet*muscle interaction: *P*≤0.001).

**Table 3:** Lipogenesis, triacylglcerol synthesis and storage genes mRNA content.

		Male			Female			Stat
		CON	HF-S	HF-FO	CON	HF-S	HF-FO	
Srebf1	EDL^†^	100 ± 5	143 ± 10*	104 ± 11	78 ± 8	103 ± 8*	104 ± 9	D, MxS
	SOL	100 ± 10^+^	99 ± 11* ^+^	92 ± 16^+^	90 ± 11	108 ± 13*	141 ± 27	
Insig1	EDL	100 ± 8	128 ± 9*	89 ± 9^#^	109 ± 15	97 ± 5*	81 ± 5^#^	D
	SOL	100 ± 8	147 ± 10*	104 ± 9^#^	112 ± 12	148 ± 18*	107 ± 16^#^	
*Dgat1* ^‡^	EDL	100 ± 15	167 ± 23	197 ± 41*	113 ± 19	178 ± 29	209 ± 22*	S, DxM
	SOL	100 ± 15	162 ± 17* ^^^	224 ± 39* ^#^ ^^^	117 ± 16	197 ± 34* ^^^	300 ± 33* ^#^ ^^^	
Scd1	EDL	100 ± 8	86 ± 8	56 ± 3* ^#^	109 ± 17	77 ± 4	84 ± 7* ^#^	DxMMxS
	SOL^†^	100 ± 11^^^	84 ± 6* ^^^	32 ± 2* ^#^	149 ± 13^^^	110 ± 19* ^^^	40 ± 5* ^#^	
Hsl	EDL	100 ± 7	128 ± 8	142 ± 14* ^#^	103 ± 12	117 ± 12	135 ± 11* ^#^	D, M
	SOL^+^	100 ± 7	117 ± 7	144 ± 13* ^#^	107 ± 12	124 ± 14	150 ± 13* ^#^	

Sterol regulatory element binding transcription factor 1 (Srebf1), insulin induced gene 1 (Insig1), diacylglycerol acyltransferase 1 (Dgat1), stearoyl-Coenzyme A desaturase 1 (Scd1) and hormone sensitive lipase (Hsl) in extensor digitorum longus (EDL) and soleus (SOL) muscles of male and female mice fed control (CON), high saturated fat (HF-S) or high fat fish oil enriched (HF-FO) diets for 14 wks (Cohort 1).

mRNA contents are expressed as a percentage of the value of male animals under CON diet. Results are mean ± SEM of 9–12 animals per group. Statistics: Two-way ANOVA: Effect of diet (D):

*P≤0.05, vs CON;

^#^ *P*≤0.05, compared to HF-S. Effect of muscle type (M):

^+^ *P*≤0.05 EDL vs SOL. Effect of sex (S):

^‡^ *P*≤0.05, male vs female. Muscle*sex interaction (MxS):

^†^ *P*≤0.05, male vs female (of same muscle). Diet*muscle (DxM):

^*P*≤0.05, EDL vs SOL (of same diet).

mRNA contents of genes involved in lipogenesis, lipolysis and triacylglycerol metabolism were mostly higher in the soleus than the EDL, but this was sex- and diet-dependent. *Srebf1* (male mice), *Hsl* (irrespective of diet or sex), *Dgat1* (HF-S, HF-FO (diet*sex) and *Scd1* (CON, HF-S (diet*muscle), male and female (sex*muscle)) exhibited greater mRNA in soleus compared to EDL muscle. Sex-dependent effects were observed for *Srebf1* (in EDL) which exhibited greater mRNA in males, and *Scd1* (in soleus) and *Dgat1* (irrespective of diet or muscle) which exhibited greater mRNA in females.

### Effect of dietary fat composition on fatty acid oxidation gene mRNA content (Table 4)

Compared to CON, HF-S decreased *Pgc1α* mRNA in the soleus muscle (diet*muscle interaction: *P*≤0.001) and *Pparα* mRNA in both muscles (effect of diet *P*≤0.05) and increased *Ampkα2* mRNA irrespective of muscle fiber type (effect of diet *P*≤0.05). Compared to CON, HF-FO increased *Cpt1b*, *Ucp3*, *Pgc1α* and *Pdk4* mRNA in both muscles (diet*muscle interaction: *P*≤0.05; *P*≤0.001; *P*≤0.001; and effect of diet: *P*≤0.001). Compared to HF-S, HF-FO increased *Cpt1b* mRNA in both muscles (diet*muscle interaction *P*≤0.05), and increased *Ppar* (effect of diet *P*≤0.05) and decreased *Ampkα2* mRNA (effect of diet *P*≤0.05), irrespective of muscle fiber type. Neither *Ampkα 1* nor *Acc-β* mRNA were changed by diet or sex (data not shown). There were no diet*sex interactions, however sex differences were observed in the mRNA content of *Cpt1b* (effect of sex: *P*≤0.001) and *Pgc1α* (effect of sex: *P*≤0.005), irrespective of muscle type, and of *Ampkα2* in the EDL only (muscle*sex interaction: *P*≤0.001).

**Table 4:** Fatty Acid Utilisation Gene mRNA Content.

		Male			Female			Stat
		CON	HF-S	HF-FO	CON	HF-S	HF-FO	
Pdk4	EDL	100 ± 18	167 ± 13*	206 ± 25*	131 ± 27	160 ± 20*	192 ± 19*	D, M
	SOL^+^	100 ± 14	176 ± 5*	234 ± 13*	118 ± 20	204 ± 28*	241 ± 24*	
Ampk1	EDL	100 ± 7	97 ± 8	101 ± 8	107 ± 12	97 ± 6	128 ± 11	M
	SOL^+^	100 ± 7	116 ± 7	118 ± 5	122 ± 8	111 ± 6	110 ± 6	
Ampk2	EDL^†^	100 ± 5	106 ± 3*	91 ± 2^#^	85 ± 5	94 ± 29*	85 ± 7^#^	D MxS
	SOL^+^	100 ± 3	103 ± 4*	93 ± 3^#^	95 ± 5	106 ± 9*	92 ± 5^#^	
Acc-	EDL	100 ± 7	110 ± 9	111 ± 10	140 ± 40	104 ± 6	104 ± 12	M
	SOL^+^	100 ± 8	122 ± 7	139 ± 6	134 ± 28	147 ± 33	154 ± 27	
*Cpt1b* ^‡^	EDL	100 ± 6	94 ± 6	115 ± 4* ^#^	65 ± 5	88 ± 3	104 ± 10* ^#^	DxMS
	SOL^+^	100 ± 4	111 ± 5	146 ± 4* ^#^	85 ± 4	89 ± 10	116 ± 9* ^#^	
*Pgc1* ^‡^	EDL	100 ± 10	88 ± 6	97 ± 5	78 ± 6	79 ± 9	94 ± 13	DxMS
	SOL	100 ± 11^^^	68 ± 9*	185 ± 33* ^#^ ^^^	87 ± 10^^^	44 ± 7*	144 ± 16* ^#^ ^^^	
Ppar	EDL	100 ± 5	84 ± 6*	90 ± 6^#^	105 ± 15	92 ± 11*	95 ± 12^#^	D, M
	SOL^+^	100 ± 9	78 ± 7*	102 ± 7^#^	104 ± 10	87 ± 6*	100 ± 8^#^	
Ucp3	EDL	100 ± 15	116 ± 7	163 ± 18*	78 ± 14	105 ± 12*	136 ± 13*	DxM
	SOL	100 ± 19^^^	196 ± 16	283 ± 25*	111 ± 27^^^	248 ± 34*	255 ± 24*	

Pyruvate dehydrogenase kinase 4 (Pdk4), AMP-activated protein kinase catalytic subunits α 1 (Ampkα 1) and α 2 (Ampkα 2), acetyl-CoA carboxylase- (Acc-), carnitine palmitoyl transferase 1b (Cpt1b), peroxisome proliferative activated receptor coactivator 1 (Pgc1), peroxisome proliferator activator receptor α (Ppar α) and uncoupling protein 3 (Ucp3) in extensor digitorum longus (EDL) and soleus (SOL) muscles of male and female mice fed control (CON), high saturated fat (HF-S) or high fat fish oil enriched (HF-FO) diets for 14 wks (Cohort 1).

mRNA contents are expressed as a percentage of the value of male animals under CON diet. Results are mean ± SEM of 9–12 animals per group. Statistics: Two-way ANOVA: Effect of diet (D):

*P≤0.05, vs CON;

^#^ *P*≤0.05, compared to HF-S. Effect of muscle type (M):

^+^ *P*≤0.05 EDL vs SOL. Effect of sex (S):

^‡^ *P*≤0.05, male vs female. Muscle*sex interaction (MxS):

^†^ *P*≤0.05, male vs female (of same muscle). Diet*muscle (DxM):

^*P*≤0.05, EDL vs SOL (of same diet).

### Effect of dietary fat composition on mitochondrial fatty acid oxidative and OXPHOS protein abundance and staining for oxidative enzymes

Compared to CON, HF-S increased CPT1b protein in the soleus muscle (diet*muscle interaction: *P*≤0.005). Compared to CON, HF-FO increased CPT1b protein in the soleus (diet*muscle interaction: *P*≤0.005) and EDL muscles (diet*muscle interaction: *P*≤0.01). Compared to HF-S, HF-FO increased CPT1b protein in the EDL muscle (diet*muscle interaction: *P*≤0.001) (Table 5). PGC1α and PPARα protein contents were unaffected by diet. Except for increased CPT1b protein in the EDL compared to soleus of female mice only (muscle*sex interaction: *P*≤0.001), CPT1b, PGC1α and PPARα proteins were not influenced by sex or muscle fibre type. Compared to CON, HF-FO increased OXPHOS ATP synthase (Complex-V) protein, responsible for the final step of mitochondrial oxidative phosphorylation (OXPHOS) (effect of diet: *P*≤0.01) (Table 5). Compared to CON and HF-FO, HF-S reduced OXPHOS Complex-III protein in the EDL muscle alone (diet*muscle interaction: *P*≤0.001, *P*≤0.005). Protein abundance of Complexes-I to-V was not sex-dependent; but was greater in the EDL than soleus (Complex-I, -II, -IV, -V, effect of muscle fibre type: *P*≤0.001; Complex-III (CON and HF-FO groups only) diet*muscle interaction: *P*≤0.001). Representative blots are featured in supporting information S1 Fig.

**Table 5:** Abundance of Fatty Acid Utilisation and Oxidative Phosphorylation Proteins.

		Male			Female			Stat
		CON	HF-S	HF-FO	CON	HF-S	HF-FO	
CPT1b	EDL	100 ± 7	74 ± 9	129 ± 19* ^#^	104 ± 18	91 ± 25	199 ± 48* ^#^	DxMMxS
	SOL	100 ± 22	183 ± 20*	233 ± 18*	43 ± 5^^^	141 ± 13* ^^^	171 ± 29* ^^^	
PGC1α	EDL	100 ± 14	107 ± 15	107 ± 16	108 ± 16	119 ± 19	158 ± 41	M
	SOL^+^	100 ± 8	100 ± 9	111 ± 7	76 ± 8	82 ± 6	102 ± 10	
PPARα	EDL	100 ± 16	106 ± 15	105 ± 16	99 ± 12	85 ± 7	117 ± 19	M
	SOL^+^	100 ± 11	78 ± 13	92 ± 16	78 ± 19	80 ± 13	87 ± 20	
Complex-I	EDL	100 ± 21	77 ± 40	68 ± 21	146 ± 43	107 ± 28	88 ± 30	M
	SOL^+^	100 ± 18	64 ± 8	33 ± 8	49 ± 10	61 ± 16	28 ± 8	
Complex-II	EDL	100 ± 10	110 ± 24	120 ± 23	136 ± 32	134 ± 22	168 ± 34	M
	SOL^+^	100 ± 15	94 ± 8	109 ± 7	88 ± 7	103 ± 10	105 ± 10	
Complex-III	EDL	100 ± 27	31 ± 7*	68 ± 13^#^	99 ± 15	35 ± 9*	94 ± 29^#^	DxM
	SOL	100 ± 17^^^	60 ± 10	53 ± 11^^^	55 ± 9^^^	60 ± 15	53 ± 11^^^	
Complex-IV	EDL	100 ± 17	87 ± 17	100 ± 15	106 ± 12	98 ± 13	114 ± 17	M
	SOL^+^	100 ± 8	104 ± 2	98 ± 8	74 ± 7	95 ± 6	101 ± 9	
Complex-V	EDL	100 ± 16	106 ± 16	108 ± 15*	106 ± 11	111 ± 14	153 ± 31*	D, M
	SOL^+^	100 ± 14	145 ± 11	148 ± 11*	69 ± 7	130 ± 8	151 ± 10*	

Carnitine palmitoyl transferase 1b (CPT1b), peroxisome proliferative activated receptor γ coactivator 1α (PGC1α), peroxisome proliferator activator receptor α (PPARα) and mitochondrial Complex-I to—V protein abundance in extensor digitorum longus (EDL) and soleus (SOL) muscles of male and female mice fed control (CON), high saturated fat (HF-S) or high fat fish oil enriched (HF-FO) diets for 14 wks (Cohort 1).

mRNA contents are expressed as a percentage of the value of male animals under CON diet. Results are mean ± SEM of 6 animals per group. Statistics: Two-way ANOVA: Effect of diet (D):

*P≤0.05, vs CON;

^#^ *P*≤0.05, compared to HF-S. Effect of muscle type (M):

^+^ *P*≤0.05 EDL vs SOL. Effect of sex (S):

^‡^ *P*≤0.05, male vs female. Muscle*sex interaction (MxS):

^†^ *P*≤0.05, male vs female (of same muscle). Diet*muscle (DxM):

^*P*≤0.05, EDL vs SOL (of same group).

**Fig 2:**
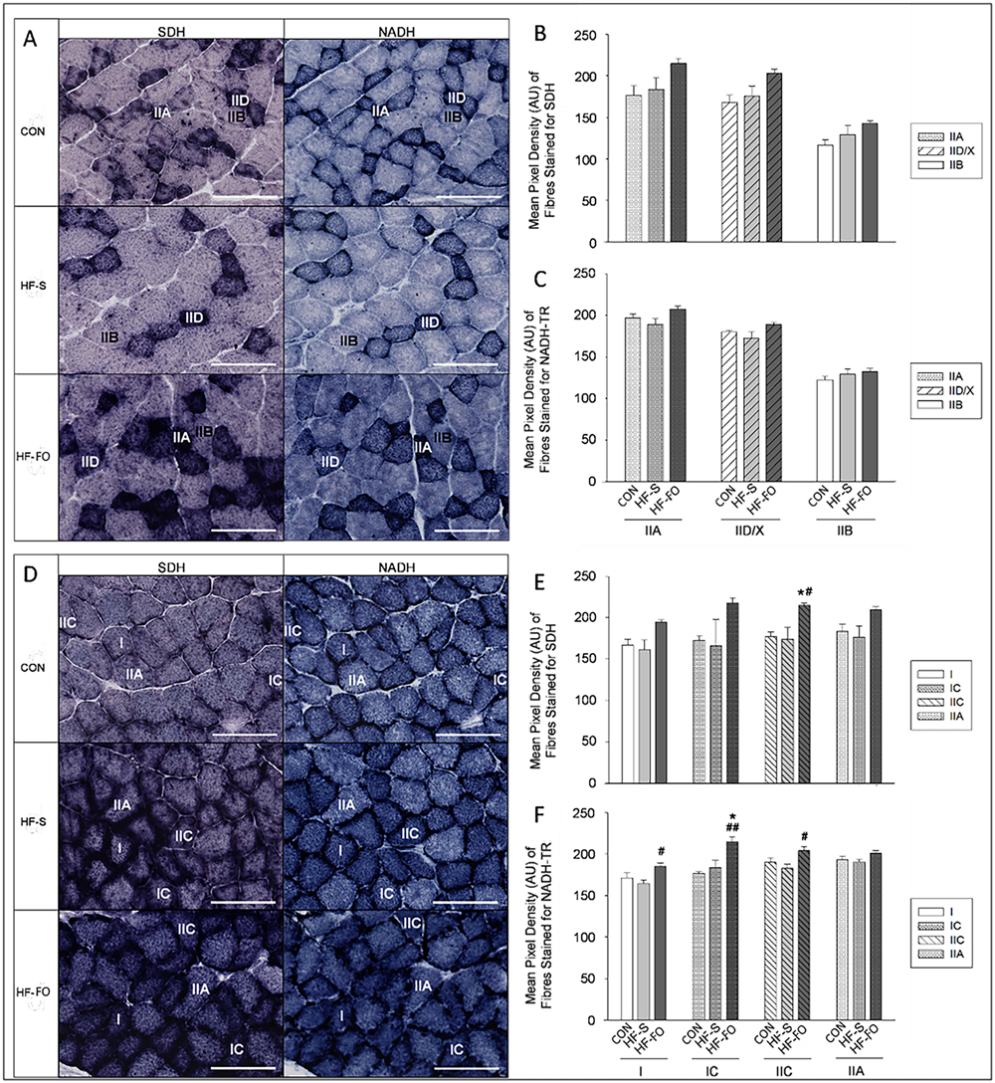
Staining for Mitochondrial Enzymes: Serial sections of the EDL (A) and soleus (D) muscle stained for SDH and NADH-TR and histological quantification SDH (B, E) and NADH-TR (C, F) staining intensity in extensor digitorum longus (B, C) and soleus (E, F) in mice fed a control (CON), high saturated fat (HF-S) or high fat fish oil enriched (HF-FO) diet for 11 wks (Cohort 2). (A, D): Scale bars represent 100 μm. n = 5/6 per group. Statistics: **(E)**: Effect of diet: *P≤0.05, compared to CON (of same fibre type); ^#^ *P*≤0.05, compared to HF-S (of same fibre type). **(F)**: Effect of diet: *P≤0.005, compared to CON (of same fibre type); ^#^ *P*≤0.05, ^##^ *P*≤0.02, compared to HF-S (of same fibre type).

Staining of SDH and NADH-TR provides a generalized readout of mitochondrial oxidative capacity. Compared to CON and HF-S, HF-FO increased soleus muscle SDH staining in a fiber type-specific manner (with type I and IIC fibers exhibiting darker staining for SDH in HF-FO muscle compared CON (IIC: *P*≤0.05 I: *P* = 0.1(trend)) and HF-S (all *P*≤0.05) muscle) (Fig. 2D, E). Compared to HF-S, HF-FO tended to increased SDH staining in type IIA fibers in the soleus muscle (*P* = 0.06). Compared to CON and HF-S, HF-FO increased NADH-TR staining in soleus type IC fibres (*P*≤0.005, *P*≤0.02, respectively) and compared to HF-S alone, HF-FO increased NADH-TR staining in the soleus muscle type I and IIC fibers (*P*≤0.05) (Fig. 2F). Compared to CON, HF-FO also tended to increase SDH and NADH-TR staining in the EDL muscle in a fiber type-specific manner, with type IID/X and IIA fibers tending to stain darker for SDH (*P* = 0.06, *P* = 0.1, respectively) and for NADH-TR (*P* = 0.09, *P* = 0.08, respectively) in the HF-FO EDL (Fig. 2A-C).

## Discussion

In this study we demonstrate that chronic consumption of a HF-S diet induces markers of intramyocellular lipid accumulation in the soleus, a predominately red or oxidative muscle, but not in the EDL, a mainly white or glycolytic muscle, in mice. Increased intramyocellular lipid specific to red muscle is consistent with fundamental fiber-specific differences in intermediary metabolism and nutrient oxidation; namely red muscle ordinarily is reliant on fat as fuel and white muscle is glucose-dependent [31]. Preferential accumulation of triacylglycerol in type I fibers has been reported in rats [39] and humans [8, 9]. Our findings also suggest that increased abundance of fatty acid transport and triacylglycerol esterification genes, and decreased expression of mediators of fatty acid oxidation in red, but not white myocytes, in response to HF-S are likely contributors to the red muscle-specific intramyocellular lipid accumulation [40–43]. While HF-S-induced changes in intermediary metabolism have in past been reported [35, 36], here we show muscle type-specificity of this effect and we highlight the importance of considering fiber type when interpreting past publications and in future study design.

In past enrichment of a high fat diet of corn oil with pharmaceutical n-3 PUFA derivative, α-ethyl DHA ethyl ester, elicited promising effects in preventing high fat diet-induced muscle triacylglycerol accretion in male C57BL/6N mice [23]. We introduce a novel finding in which enrichment of a chronic HF-S diet with natural fish oil abolished markers of intramyocellular lipid accretion in the soleus and quadriceps muscles of mice. In fact histological muscle lipids in HF-FO-fed mice was comparable visually to that observed in a low-fat standard chow-fed control group, although it is important to note that not only the fat, but also the protein and carbohydrate contents, of these diets differed. Whether this is a red fiber type-predominant phenomena requires investigation by quantifying intramyocellular lipid in serial sections with individual fiber type determination. n-3 PUFAs, especially of marine origin, have long been hailed as effective mediators of hyperlipidemia [22, 23, 44–46] and at preventing hepatocellular lipid accumulation [47] and have therefore gained popularity as an adjunct clinical therapeutic. Based on past literature, the mechanism for the observed muscle lipid-lowering effect is unclear; just a handful of studies [24–30, 48] provide disparate findings on the effect of dietary n-3 PUFA enrichment on muscle lipid metabolism. We therefore sought to determine the potential mechanisms by which partial replacement of dietary fat with fish oil in a HF-S setting prevent lipid accumulation in mouse skeletal muscle. One of the main confounders encountered when assessing past published studies for evidence of a mechanism for n-3 PUFAs preventing intramyocellular lipid/triacylglycerol was that direct comparison between studies was not possible. This is because diets of varying length, energy intake from fat, proportion of n-3 PUFAs incorporated, n-3 PUFA species composition and n-3 PUFA source (marine oil, concentrates, ethyl esters) were implemented, as were varying rodent strain and gender. Another notion neglected was the effect of HF-FO in red muscle since the majority have assessed muscles of predominantly a white fast-twitch glycolytic nature [24–30]. In order to assess potential mechanisms, we therefore undertook a single-study analysis of mediators of lipid metabolism pathways in both red and white muscle to provide a snap-shot of the overall lipid metabolism and importantly to prevent inter-study variation.

Prevention of markers of intramyocellular lipid accretion in the red muscle from HF-FO mice, along with a congruent expression profile of lipid metabolism genes in both red and white muscle types, suggests that n-3 PUFAs are protective irrespective of muscle fiber type. This is, however, in contrast to a past report [48], previously unprecedented in its investigation of diet-induced effects of fish oil or lard feeding on red and white muscle contractile and metabolic gene abundance. While there were several diet-by-muscle interactions reported, dietary fat predominantly elicited responses in the white EDL muscle [48]. The source of disparity between their white muscle-specific findings and our fiber type-independent responses are likely a result of acute [48] versus chronic intervention and due to consumption of a relatively low fat diet [48] versus an “extreme” high fat diet.

We hypothesised that the mechanisms through which dietary fish oil enrichment ameliorates HF-S-induced intramyocellular lipid are via increased muscle fatty acid uptake in conjunction with upregulated fatty acid disposal and restrained lipogenesis. The observation that HF-FO elicited a co-ordinate increase in the mRNA content of 3 skeletal muscle fatty acid transporters (*Fat/Cd36*, *Fatp1*, *Fatp4*) supports this hypothesis. Our finding is also in keeping with previous research in which FAT/CD36 and fatty acid uptake increased in the white muscle of fish oil high fat-fed mice (35.5% E fat; vs tallow) [24] and in myotubes exposed to EPA (0.6 mM for 24 h) [49]. We believe that increased lipid uptake by skeletal muscle in a HF setting may be a compensatory response to prevent hyperlipidemia. We hypothesise that uptaken fatty acids are then channelled into disposal pathways and the fate of surplus fatty acids in muscle therefore warranted investigation. Induction of *Pdk4* mRNA is consistent with dietary fat triggering a switch from carbohydrate to fatty acid oxidation in an environment of fatty acid surplus [50, 51]. However, in past, HF-FO in female Wistar rats completely prevented [52] (47% E fat lard-diet vs equi-caloric diet 7% lard replaced with marine oil), and in humans partially ameliorated (75% fat vs equi-caloric diet 15% fat replaced with n-3 PUFA), induction of PDK4 activity [53]. To exert their full effect, n-3 PUFAs require tissue incorporation, a process which takes ~4 months to reach steady-state [54]. We believe the mentioned short [52] or acute [53] interventions may have been insufficient for n-3 PUFAs to elicit their full effects, in comparison to our diet of chronic duration, at least 3 times longer than past reported diets [52, 53]. This suggests that traditional pathways of fatty acid disposal are switched on by HF-FO. Traditionally DGAT1 is considered an essential intracellular enzyme in preventing harmful lipid intermediate accumulation in tissues by ensuring non-oxidised fatty acids enter neutral triacylglycerol storage [42]. In this study, parallel increases in *Dgat1* and lipolytic *Hsl* also suggest that HF-FO may provide an alternative disposal route by promoting a futile triacylglycerol-fatty acid cycle [55]. This simultaneous lipolysis and re-esterification to triacylglycerol would promote energy consumption, limiting triacylglycerol synthesis in myocytes and preventing intramyocellular triacylglycerol accretion. This is the first evidence to date that HF-FO may promote futile cycling, but this requires testing at the substrate level. An increase in mitochondrial fatty acid transporter, CPT1 [56], the rate-limiting step in fatty acid oxidation [57], may actively prevent fatty acid from entering esterification and storage [58], providing further evidence for conventional fatty acid disposal pathways being triggered by HF-FO. The interaction of transcription co-activator PGC1α and transcription factor PPAR α is known to enhance fatty acid oxidation [59]. The increase in *Pgc1* α mRNA and prevention of HF-S-induced *Ppar* α suppression by HF-FO is consistent with beliefs that n-3 PUFAs are natural PPAR α agonists and that n-3 PUFAs, via PPAR α, exert triacylglycerol-lowering effects directing fat away from storage into oxidation [60]. Although no change in their respective protein contents were observed, measures of *Pgc1* α mRNA are not necessarily reflected at the protein level [61, 62]. Mechanistically, greater *Ppar* α and *Pgc1* α mRNA combined with CPT1b mRNA and protein induction by HF-FO may promote mitochondrial fatty acid entry and oxidation, quenching intramyocellular triacylglycerol. In order to understand the contribution of mitochondrial OXPHOS in disposal pathways, we measured the protein abundance of OXPHOS Complexes I-V, as they reflect mitochondrial density and oxidation [63]. Despite research investigating the effect of HF-FO (60% E fish oil) on OXPHOS proteins in murine liver [29], to the best of our knowledge, this is the first characterisation of HF-FO-mediated changes in OXPHOS Complex I-V protein in muscle. HF-FO induction of Complex-V reflects increased flux through this pathway and greater availability of substrate from fatty acid oxidation. Increased mitochondrial enzymes, SDH and NADH-TR, in HF-FO, particularly in red muscle, are also suggestive of mitochondrial oxidative phosphorylation contributing to the prevention of intramyocellular lipid accretion. Though the contribution of peroxisomal pathways of fatty acid disposal requires investigation.

Finally, our study was designed to investigate the effects of HF-FO in both males and females, as past studies have reported sex-specific responses to HF feeding in rodents [35, 36]. In contrast, we demonstrated that both sexes were equally vulnerable to HF-S-induced metabolic dysfunction at the whole-body and skeletal muscle levels. In past, HF-S females upregulated adipose storage and muscle fatty acid oxidation [36]; whereas males, less efficient at promoting these pathways instead directed triacylglycerol to the liver, where oxidation increased [36]. Though, multiple methodological factors, including diet composition (31.4% [36] vs 65% saturated fat), duration (6 [36] vs 3.5 months) and species (rats [36] vs mice) may produce disparities. In keeping with HF-S eliciting no sex-specific responses, HF-FO was equally protective in males and females in the current study. These data suggest that gender-specific responses to n-3 PUFA could be a trivial consideration in the clinical setting and in future studies.

In conclusion, this is a novel study identifying the potential mechanisms by which n-3 PUFA enrichment, from fish oil, may prevent HF-S-induced dysfunction in fatty acid metabolism pathways in skeletal muscle, and is the first to investigate both the effects in red and white muscle, and female and male settings. We provide insights into the changes induced within skeletal muscle at the mRNA and protein levels as a result of dietary fatty acid composition and implicate increased fatty acid transport, enhanced myocyte triacylglycerol storage and synthesis, and reduced/incomplete oxidation in the inappropriate intramyocellular lipid accretion observed in red oxidative muscle upon long-term HF-S feeding. According to analyses of the mRNA, protein and enzyme contents of key mediators of fatty acid metabolism, we demonstrate that HF-FO likely prevents HF-S-induced intramyocellular lipid accumulation by a concurrent increase in mediators of fatty acid uptake and utilisation, and suppression of mediators promoting fatty acid storage and lipogenesis. Future research would best focus on confirming the proposed mechanistic pathways at the substrate level. Furthermore given the benefits of dietary fish oil enrichment in preventing lipid-induced metabolic dysfunction, understanding the translatability of these findings to a clinical setting is vital, especially as 60% E from fat is an “extreme” high fat diet whilst replacing 7.5% of saturated fat with n-3 PUFAs from fish oil is the equivalent of a high intake of n-3 PUFAs in humans.

## Supporting Information

S1 Fig Representative Western Blots Probed for Fatty Acid Utilisation and Oxidative Phosphorylation Proteins. Carnitine palmitoyl transferase 1b (CPT1b), peroxisome proliferative activated receptor γ coactivator 1 α (PGC1 α) and peroxisome proliferator activator receptor α (PPAR α) and Complex-I to—V protein in the extensor digitorum longus (EDL) and soleus (SOL) muscles of male and female mice fed a control (CON), high saturated fat (HF-S) and high fat fish oil enriched (HF-FO) diet for 14 wks (Cohort 1).
(TIF)

S1 Table The sequence, assigned product size and optimised concentration of primers used to determine mRNA content of genes of interest in skeletal muscle. (DOCX)
