# Supplementary figures and images for: Dietary Enrichment with Fish Oil Prevents High Fat-Induced Metabolic Dysfunction in Skeletal Muscle in Mice
Source: PLoS One. 2015 Feb 6;10(2):e0117494. doi: 10.1371/journal.pone.0117494 (PMC4320112; doi:10.1371/journal.pone.0117494)

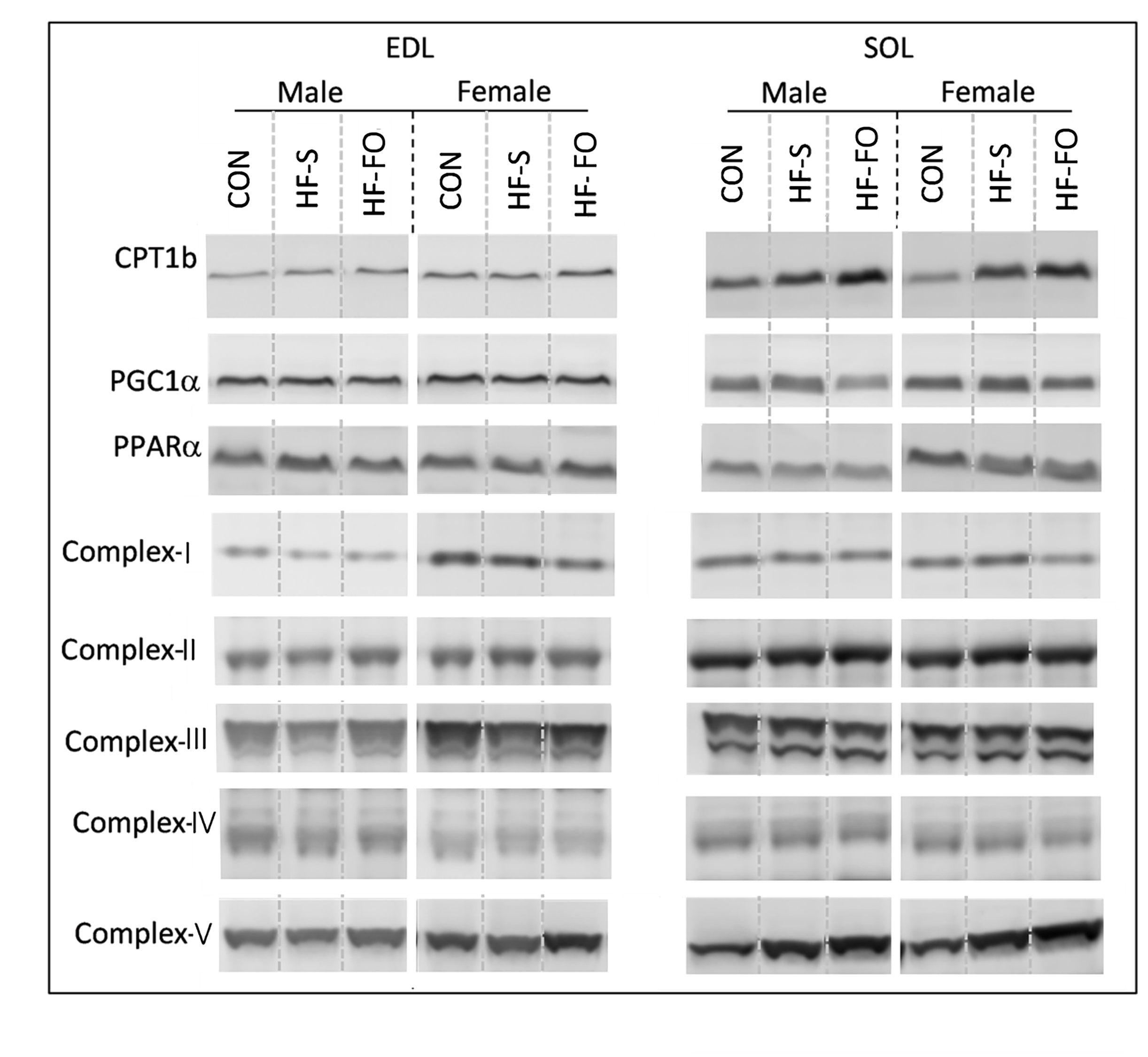

Supplement: S1 Fig — Carnitine palmitoyl transferase 1b (CPT1b), peroxisome proliferative activated receptor γ coactivator 1 α (PGC1 α) and peroxisome proliferator activator receptor α (PPAR α) and Complex-I to—V protein in the extensor digitorum longus (EDL) and soleus (SOL) muscles of male and female mice fed a control (CON), high saturated fat (HF-S) and high fat fish oil enriched (HF-FO) diet for 14 wks (Cohort 1). (TIF) [file pone.0117494.s001.tif]
