# Supplementary material for: Dietary Enrichment with Fish Oil Prevents High Fat-Induced Metabolic Dysfunction in Skeletal Muscle in Mice
Source: PLoS One. 2015 Feb 6;10(2):e0117494. doi: 10.1371/journal.pone.0117494 (PMC4320112; doi:10.1371/journal.pone.0117494)
Supplement: S1 Table — (DOCX) [file pone.0117494.s002.docx]

| **S1 Table.** The sequence, assigned product size and optimised concentration of primers used to determine mRNA content of genes of interest in skeletal muscle. | | | |
| --- | --- | --- | --- |
| Gene  (Accession number) | Forward (FWD) primer sequence (5’-3’)  Reverse (REV) primer sequence (5’-3’) | Size (bp) | [Primer] reverse mix (nM) |
| *Dgat1*  (NM_010046) | GATCTGAGGTGCCATCGTCT  GGATCAGCATCACCACACAC | 137 | 125 |
| *Pdk4*  (NM_013743) | CCATGAGAAGAGCCCAGAAG  ATGCCTTGAGCCATTGTAGG | 145 | 16.63 |
| *Scd1*  (NM_009127) | CTATGGATATCGCCCCTACG  TAGTCGAAGGGGAAGGTGTG | 151 | 16.63 |
| *Slc27a4 (Fatp4)* (NM_011989) | CAGTGAGATGGCCTCAGCTA  TCCAGAAGAGGGTCCAGATG | 166 | 500 |
| *Slc27a1 (Fatp1)* (NM_011977) | GCAGGTACTACCGCATTGCT  AACCCGTAGATGACGCACTG | 173 | 500 |
| *Insig1*  (NM_153526) | GACGAGGTGATAGCCACCAT  TGGCCCATTCTCTCTTGAAC | 179 | 500 |
| *Acc-β*  (NM_133904) | TGGAGTCCATCTTCCTGTCC  GGACGCCATACAGACAACCT | 186 | 125 |
| *Ucp3*  (NM_009464) | GATGTGGTGAAGGTCCGATT  GGCATTTCTTGTGATGTTGG | 193 | 31.25 |
| *Ampkα2* (NM_178143) | TGATCAGCACTCCGACAGAC  TCAGGTCCCTATGGACAACC | 214 | 500 |
| *Polr2c* (NM_009090) | TGAGGTGCAATGAAGACCAG  CTTGGCATAGGCTCGAAGTC | 222 | 250 |
| *Srebf1* (NM_011480) | GCAGTCTGCTTTGGAACCTC  GAAGCAGCAAGATGTCCTCC | 242 | 500 |
| *Fabp_pm_* (NM_010325) | GCGGTTTTGACTTCTCTGGA  CCAGGCATCCTTATCACCAT | 255 | 62.5 |
| *Cpt1b*  (NM_009948) | GGTCGCTTCTTCAAGGTCTG  AAGAAAGCAGCACGTTCGAT | 270 | 16.63 |
| Lipe (*Hsl*) (NM_001039507) | GGAACTAAGTGGACGCAAGC  TTGACATCAGAGGGTGTGGA | 277 | 500 |
| *Fat/Cd36* (NM_007643) | GCTCTCCCTTGATTCTGCTG  TGGGTTTTGCACATCAAAGA | 286 | 125 |
| *Tbp*  (NM_013684) | GGACCAGAACAACAGCCTTC  GTGGGTTGCTGAGATGTTGA | 298 | 500 |
| *Rplp0*  (NM_007475) | GCATCACCACGAAAATCTCC  TACCCGATCTGCAGACACAC | 305 | 250 |
| *Pgc1α*  (NM_008904) | GTACAACAATGAGCCTGCGA  AGTGCTAAGACCGCTGCATT | 332 | 500 |
| *Pparα* (NM_001113418) | ACGATGCTGTCCTCCTTGAT  TCATCTGGATGGTTGCTCTG | 339 | 500 |
| *Ampkα1* (NM_00103367) | AGCCGACTTTGGTCTTTCAA  ATCTTTTATTGCGGCCCTCT | 356 | 1,500 |
| Above primer sequences were flanked by universal sequence at 5’ end: FWD = aggtgacactatagaata; REV= gtacgactcactataggga. | | | |
